# The effect of action contingency on social perception is independent of person-like appearance and is related to deactivation of the frontal component of the self-agency network

**DOI:** 10.1038/s41598-022-22278-x

**Published:** 2022-10-15

**Authors:** Yumi Hamamoto, Yukiko Takahara, Kelssy Hitomi dos Santos Kawata, Tatsuo Kikuchi, Shinsuke Suzuki, Ryuta Kawashima, Motoaki Sugiura

**Affiliations:** 1 Institute of Development, Aging and Cancer, Tohoku University, Seiryo-Machi 4-1, Aoba-Ku, Sendai, 980-8575 Japan; 2 School of Medicine, Tohoku University, Sendai, Japan; 3 Frontier Research Institute for Interdisciplinary Science, Tohoku University, Sendai, Japan; 4 Centre for Brain, Minds and Markets, Department of Finance, Faculty of Business and Economics, The University of Melbourne, Melbourne, Australia; 5 International Research Institute of Disaster Science, Tohoku University, Sendai, Japan; 6 Present Address: NB155 CoCo, Northumberland Building, Department of Psychology, Northumbria University, Newcastle Upon Tyne, NE1 8ST UK

**Keywords:** Agency, Perception

## Abstract

The detection of object movement that is contingent on one’s own actions (i.e., movements with action contingency) influences social perception of the object; such interactive objects tend to create a good impression. However, it remains unclear whether neural representation of action contingency is associated with subsequent socio-cognitive evaluation of “contacting agents”, or whether the appearance of agents (e.g., face- or non-face-like avatars) is essential for this effect. In this study, we conducted a functional magnetic resonance imaging (fMRI) task with two phases: contact (contact with face- or non-face-like avatars moving contingently or non-contingently) and recognition (rating a static image of each avatar). Deactivation of the frontoparietal self-agency network and activation of the reward network were the main effects of action contingency during the contact phase, consistent with previous findings. During the recognition phase, static avatars that had previously moved in a contingent manner deactivated the frontal component of the frontoparietal network (bilateral insula and inferior-middle frontal gyri), regardless of person-like appearance. Our results imply that frontal deactivation may underlie the effect of action contingency on subsequent social perception, independent of person-like appearance.

## Introduction

Various actions occur during social interactions, such as nodding, backchannelling, and facial expressions; these non-verbal actions influence the perception of others. Suppose that we encounter an unknown tribesperson, animal, or object while walking in the jungle. If the agent moves in response to our movement, we will assume that it is interested in us; in turn, this will cause us to be interested in the agent. Conversely, if the agent does not respond or moves in a manner unrelated to our movement, we will assume that it has no interest in us, and thus in turn have no interest in it. An important determinant of the social perception of an agent is whether its movements are contingent on our actions, i.e., action contingency^1–4^. If the movements of the agent are temporarily and semantically contingent on our action (e.g., the agent looks in the direction that we point in), we should perceive intention from the agent and form an impression toward it as a target of social interaction, but not if its actions are non-contingent to ours.

Several behavioral studies have provided empirical evidence of the effect of action contingency on social perception (i.e., perceiving a contacting partner as a target of social interaction and having feelings of familiarity and so on)^5–9^. These studies showed that individuals have a stronger preference for a partner who mimics their actions^5,6^. Action synchrony (e.g., producing the same rhythm as another person while drumming) also increases liking for a contacting partner^7–9^. Considering that partners that mimic or synchronize with an individual’s action move contingently in accordance with that individual’s action, these findings can be explained by the effect of action contingency on social perception.

There is a view that attempts to understand the effect of action contingency on social perception as an extension of the perception of self-agency in action^10^; an influential model posits that action contingency plays a key role in the sense of self-agency^4,11–13^. According to this “forward model”, when an individual executes an action, commands are sent to the motor system^14^, and sensory feedback from bodily movements (e.g., somatosensory, visual, or auditory inputs) is provided based on the commands and an internal model of the body and physical environment^4,15^. Self-agency is established when predicted sensory input matches actual sensory input^11–13^. For example, in the case of hand clapping, the corresponding sound is predicted; when the actual sound matches that predicted, it is attributed to one’s own action, and the individual knows that they have clapped. In addition to research on self-awareness, research on schizophrenia draws on this self-agency system; malfunction therein may explain disturbed perception of the self, which is the core pathology of schizophrenia^4,16–18^. Some researchers have proposed that recognition of others is explained by the forward model^10,19,20^, despite temporal and semantic “looseness” in action contingency during social interactions compared with self-agency. This implies that the existence of other interactive targets is recognized when sensory input is detected that is not directly derived from, but can be predicted based on, one’s own actions. For example, in the case of an interaction between a mother and infant, when the mother moves based on the infant’s action, the infant assumes that the mother’s action is contingent on its own action, even though it does not match the predicted sensory input. Thus, actions by the mother are associated with, but not attributed to, the infant’s own action, which leads to the detection of targets for social interaction. In fact, at 3 months of age, infants start to show a preference for targets that mimic their own actions^10^.

Previous studies on the effect of action contingency on the impressions of others evaluated two main underlying neural mechanisms. Several functional magnetic resonance imaging (fMRI) studies have supported the hypothesis that action contingency is an extension of self-agency^21–23^. These previous fMRI studies investigated brain activation during contact with contingent or non-contingent targets; a contingent target (but not a non-contingent target) demonstrated a rhythm similar to a participant’s rhythm, and frontoparietal regions were deactivated during contact with the contingent target^21–23^. This deactivation of the frontoparietal network, which is composed of the superior, middle, and inferior frontal gyri, insula, temporoparietal junction, and superior temporal gyri, involves temporal regions and contributes to the detection of self-agency based on action contingency; frontoparietal regions are deactivated when participants detect feedback contingent on their own actions^4,24–28^. Therefore, frontoparietal deactivation during contact with contingent targets implies the involvement of self-agency.

Other studies reported findings outside the framework of self-agency^21,23^. These studies also used rhythm-based tasks involving contingent and non-contingent partners, and found that the degree of liking after contact was correlated with brain activity, for example in the ventromedial prefrontal cortex^21^ and caudate^21,23^. These brain regions participate in reward processing^29–31^. An association with reward processing is consistent with the notion that contingent feedback is processed as a reward^32,33^, which implies that improvements in social perception after contact reflect a sense of reward conferred by action contingency.

However, two issues related to this topic have not been satisfactorily explored. The first is the level of the effect, i.e., whether action contingency influences social perception at the level of the contacting partner’s representation. Previous fMRI studies^21–23^ investigated neural activation only during contact, and demonstrated that it related to encoding of a contacting partner; however, it remains unclear whether observed neural activation (i.e., deactivation in frontoparietal regions and activation in reward-related regions) influences social perception after contact as a representation of the contacting partner. When a specific experience is encoded as a specific attribution, the neural network activated during the experience is also activated during recognition; for example, just imagining a specific motion activates corresponding motor-related brain regions without actual motion^34^, and merely looking at static images of specific tools (e.g., scissors or a hammer) will activate brain regions related to corresponding specific motions (e.g., opening and closing the blades of the scissors)^35^. If the action-contingency effect is encoded as the contacting partner’s representation, neural activation related to action contingency during contact should be replicated during recognition. If brain activation during contact is not replicated during recognition then action contingency does not directly represent neural activation of social perception.

The second issue is the influence of an object’s appearance, that is, whether a person-like appearance is essential to establish the action-contingency effect on social perception. Investigation of whether a person-like appearance influences the action-contingency effect has important implications regarding the cognitive and developmental processes of the action-contingency effect because a person-like appearance is also an important characteristic that induces social perception. However, the relationship between the action-contingency effect and person-like appearance has been little studied. The main theory posited by developmental psychology studies is that a person-like appearance is a prerequisite for the action-contingency effect^36–39^. These studies showed that, even a few hours after birth, newborn infants can detect person-like eyes and faces, and show a preference for objects with a person-like appearance^36–39^; this demonstrates that the detection of person-like appearance is innate. Some studies have reported that a preference for person-like appearance precedes that for contingent object motion^10,40,41^, and the mainstream developmental studies logically imply that the action-contingency effect arises from the effect of person-like appearance on social perception. On the other hand, other studies argue that the action-contingency effect independently influences social perception^2,10,42–44^, given that both infants and adults show a preference for objects that move contingent on their actions compared to objects moving non-contingently, even if the object does not have a person-like appearance^2,10,42–44^. Determining whether the action-contingency effect is independent of the effect of person-like appearance would help to explain how we recognize the existence of others, and may also promote the development of interactive devices, such as social robots, as well as artificial-intelligence-based counselling applications. However, previous fMRI studies^21–23^ did not compare action-contingency effects on social perception with-versus-without a person-like appearance.

**Figure 1:**
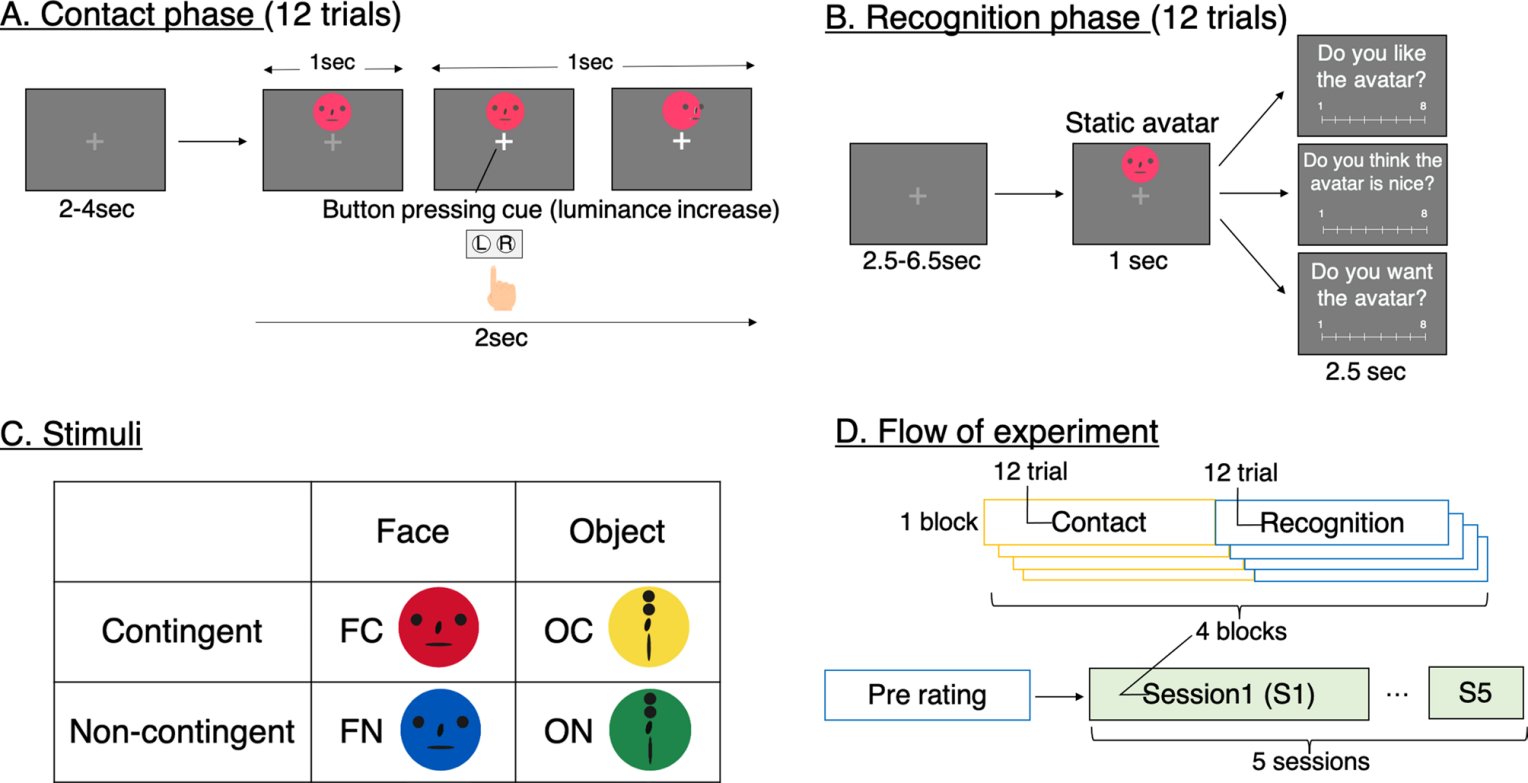
Experimental design and stimuli. (**A**) Design of the contact phase. Participants pressed the right or left button when the fixation point became brighter. The avatars turned left or right; the timing and direction of turning differed by condition. (**B**) Design of the recognition phase. Participants observed the static avatars and rated them by pressing the button. Participants answered three questions: “Do you like the avatar?”, “Do you think the avatar is nice?”, and “Do you want the avatar?”. (**C**) The four avatar conditions. We presented the four avatar types, each representing a different condition, and used a 2 × 2 design (2 contingency factors × 2 face perception factors): face contingent (FC), face non-contingent (FN), object contingent (OC), and object non-contingent (ON). We drew the four avatars in different colors (red, blue, yellow, and green) to ensure that participants could identify the avatar type. (**D**) Sequence of the functional magnetic resonance imaging (fMRI) experiment. In total, 12 trials of the contact and recognition phases comprised one block, and four such blocks comprised one session. Participants completed five sessions in an MRI scanner. Before each session, participants rated the avatars (“Pre rating” in **D**); these ratings represent the baseline scores.

To address these two issues related to the neural correlates of the action-contingency effect on social perception, we conducted an fMRI study that investigated neural representations during recognition and the influence of a face-like appearance. We created face- and non-face-like avatar stimuli, and conducted an fMRI task with two phases: contact (Fig. 1A) and recognition (Fig. 1B). In the contact phase, participants interacted with avatars by pressing a button; the avatars moved contingently or non-contingently in response to a participant’s button press. Then, in the recognition phase, static images of the same avatars as used in the contact phase were presented and participants indicated their degree of liking for each. The experiment used a 2 × 2 design (2 contingency factors × 2 face-perception factors) and four avatars represented different conditions (Fig. 1C): a face-like avatar moving contingently, a face-like avatar moving non-contingently, a non-face-like avatar (i.e., object) moving contingently, and a non-face-like avatar moving non-contingently (FC, FN, OC, and ON, respectively).

To address the first issue, we first investigated brain activity during the contact phase and then examined whether this activity was replicated during the recognition phase. Comparison of neural activation during contact with contingent avatars (FC and OC) with that during contact with non-contingent avatars (FN and ON) provided a measure of neural activity related to the detection of each avatar’s action contingency. If activity related to action contingency during the contact phase is observed during the recognition phase, the avatar’s action contingency can be considered to be encoded as a representation of the avatar; this information will then inform social perception of the avatar. By contrast, if the action-contingency-related neural activity observed during the contact phase is not observed during the recognition phase, representations of the avatar (in terms of its action contingency) will not influence social perception of the avatar. We also investigated the correlation between the neural activity representing the effect of action contingency on social perception during the recognition phase and subjective liking scores.

To address the second issue, we investigated whether a person-like appearance is needed to detect neural activity representing the action-contingency effect on social perception. If the action-contingency effect is dependent on a person-like appearance, then brain activity related to the effect of action contingency on social perception should be observed during the recognition phase when investigating the main effect of face (e.g., greater brain activation when looking at face-like than non-face-like avatars; [FC + FN] > [OC + ON]), as well as an interaction between the face and contingency effects (e.g., an interaction of an avatar’s face-like appearance with action-contingency-enhanced brain activation; [FC – OC] > [FN – ON]), rather than brain activation representing the main effect of contingency (e.g., greater brain activation when looking at contingent than non-contingent avatars; [FC + OC] > [FN + ON]). Conversely, if the action-contingency effect is independent of person-like appearance, brain activity related to the effect of action contingency on social perception during the recognition phase should be observed as the main effect of contingency.

## Results

### Behavioral data: liking scores

**Figure 2:**
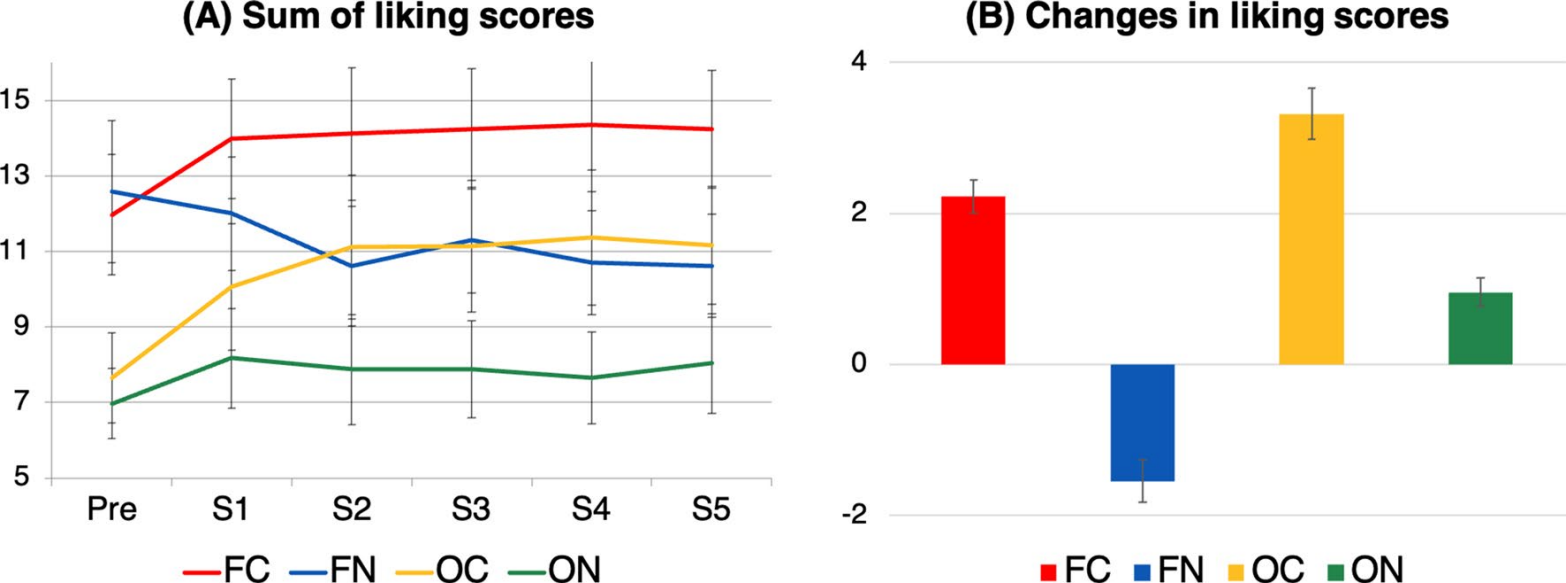
Participants’ mean liking scores before and at the end of the experiment. (**A**) Liking scores for each condition and session [from session 1 (S1) to session 5 (S5)], as well as before the experiment (Pre). The liking score was calculated as the sum of three questionnaire item scores. The participants’ mean liking scores are presented graphically; error bars represent 95% confidence intervals (CIs). (**B**) Changes in liking scores for each condition. Changes in liking scores were calculated as the difference between the pre-experiment and mean liking scores for S1–S5 (i.e., [(S1 + S2 + S3 + S4 + S5)/5] − [Pre]). Error bars represent 95% CIs.

We expected that the social perception of the participants would gradually improve during the experiment, and investigated the effects of action contingency (two within-subject levels: contingent and non-contingent), face perception (two within-subject levels: face and object), and session (five within-subject levels: 1–5) using repeated-measures three-way analysis of variance (ANOVA). Unexpectedly, participants showed an action-contingency effect beginning in the first session (Fig. 2A) with no significant main effect of session [F(4, 120) = 0.22, *P* = 0.93, η^2^ = 0.0002 (95% confidence interval [CI] 0, 0.0003)] and a significant interaction between session and contingency [F(4, 120) = 3.35, *P* = 0.01, η^2^ = 0.004 (95% CI 0.0008, 0.01)], whereas the interaction between session and face perception was marginally significant [F(4, 120) = 2.20, *P* = 0.07, η^2^ = 0.004 (95% CI 0.0003, 0.003)]. We conducted post hoc analysis to evaluate the interaction between session and contingency, and found a significant effect of session in the non-contingent condition [F(4, 120) = 2.54, *P* = 0.04, η^2^ = 0.007 (95% CI 0.0008, 0.02)] but not in the contingent condition [F(4, 120) = 1.52, *P* = 0.20, η^2^ = 0.004 (95% CI 0.0003, 0.01)]. Then, we conducted multiple comparisons using Shaffer’s method to investigate the effects of session on the non-contingent group; there was no significant effect. Also, there was no significant interaction among contingency, face perception, and session [F(4, 120) = 0.42, *P* = 0.79, η^2^ = 0.0002 (95% CI  0, 0.0005)].

To investigate the action-contingency effect on subjective liking ratings during the recognition phase, we conducted a two-way ANOVA with a 2 × 2 design (2 contingency factors × 2 face-perception factors) (Fig. 2B). We detected significant main effects of contingency [F(1, 30) = 19.5, *P* < 0.001, η^2^ = 0.13 (95% CI 0.06, 0.20)] and face perception [F(1, 30) = 4.82, *P* = 0.04, η^2^ = 0.04 (95% CI 0.001, 0.11)]. There was no significant interaction between contingency and face perception [F(1, 30) = 3.58, *P* = 0.07, η^2^ = 0.007 (95% CI 0.0001, 0.03)]. These results imply that action contingency influences social perception in a manner independent of person-like appearance.

### Behavioral data: reaction time and button pressing preference

To exclude the possibility that the behavioral and fMRI results were derived from condition-specific biases in button pressing or differences in task difficulty, we investigated the participants’ button-pressing preferences during the contact phase (i.e., how frequently they pressed the right-hand button compared to the left-hand button during the experiment) and their button-pressing reaction times during the contact and recognition phases. Two-way ANOVA showed that button-pressing preference during the contact phase did not significantly differ between the conditions. There was no significant main effect of contingency or face perception, or interaction between these factors. There was also no significant difference in the numbers of failed trials in each condition. Finally, the main effects and interaction for each condition were not significant.

Reaction times during the contact phase showed a significant main effect of face perception [F(1, 1594) = 6.78, *P* = 0.009, η^2^ = 0.0008 (95% CI 0, 0.002)], implying that reaction time was longer toward face avatars than toward non-face avatars. There was no significant main effect of contingency or significant interaction between contingency and face perception. Reaction times during the recognition phase showed no significant main effect of contingency or face perception, although the interaction was significant [F(1, 2551) = 31.5, *P* < 0.001, η^2^ = 0.002 (95% CI 0.0008, 0.004)]. Post hoc analysis showed that reaction times under the contingent (vs. non-contingent) condition were shorter for faces (*P* < 0.001) and longer for objects (*P* = 0.009). See Supplementary Table 1 for further details.

### fMRI data

**Figure 3:**
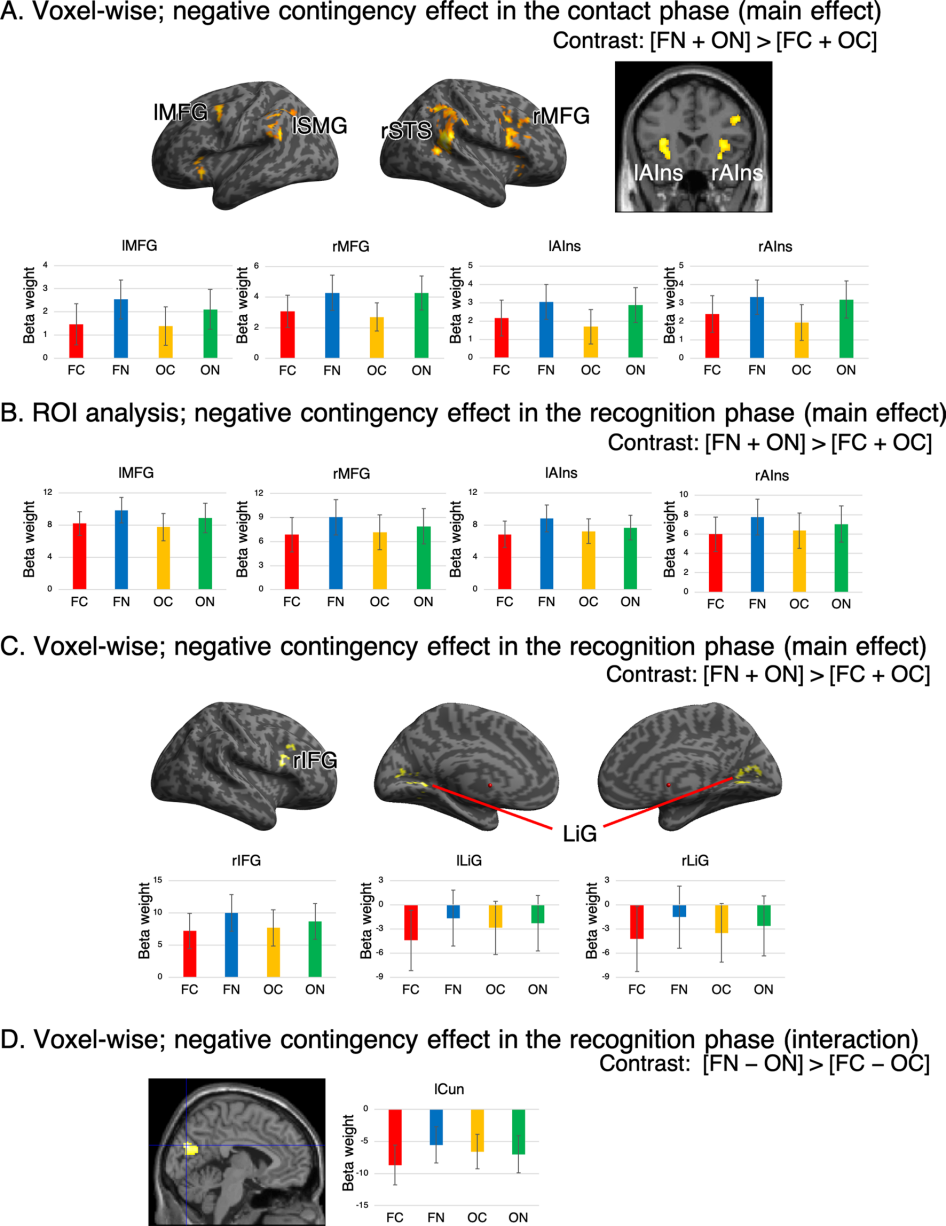
Brain activation representing a negative contingency effect. (**A**) Brain activation during the contact phase representing a negative contingency effect according to voxel-wise analysis. The threshold was set according to an uncorrected *P-*value < 0.001 and corrected *P-*value < 0.05 based on cluster size. The graphs are activation profiles (beta weights) for the middle frontal gyrus and bilateral anterior insula, which were significantly activated in both voxel-wise and region of interest (ROI) analyses. Error bars represent 95% CIs. (**B**) Activation profiles of brain regions in the bilateral middle frontal gyri and bilateral anterior insula during the recognition phase. Error bars represent 95% CIs. (**C**) Brain activation during the recognition phase according to voxel-wise analysis, derived from the main effect of the negative contingency effect contrast. (**D**) Brain activation during the contact phase, derived from the contrast representing the negative contingency effect of the interaction between contingency and face perception (i.e., [FN − ON] > [FC − OC]). lSMG = left supramarginal gyrus; rSTS = right superior temporal sulcus; rIFG = right inferior frontal gyrus; LiG = lingual gyrus.

Analysis of the negative contingency effect (i.e., activation in the [FN + ON] > [FC + OC] contrast) during the contact phase showed a significant main effect of contingency. The left supramarginal gyrus and right superior temporal sulcus cluster expanded to the supramarginal gyrus, the bilateral middle frontal gyri cluster expanded to the inferior frontal gyrus in the right hemisphere, and the bilateral anterior insula deactivated in the contingent compared with the non-contingent condition (Table 1, Fig. 3A,B). The aforementioned results are consistent with findings from previous studies, which showed frontoparietal deactivation during contact with contingent targets^22,23^. We did not observe any significant interaction in terms of activation (i.e., [FN − ON] > [FC − OC] contrast), which implies that the action-contingency effect was independent from person-like appearance.

**Table 1:** Brain regions with a negative contingency effect and results of region of interest (ROI) analysis.

Anatomical label	MNI coordinates (peak)				t-value	Cluster			
	L/R	x	y	z		Size (voxels)	Corrected p-value		
Supramarginal gyrus	L	− 54	− 48	28	4.80	613	< 0.001		
Superior temporal sulcus	R	52	− 44	10	6.30	1405	< 0.001		
Middle frontal gyrus	L	− 36	4	56	4.78	267	0.004		
	R	36	8	32	5.13	828	< 0.001		
Anterior Insula	L	− 32	22	6	4.73	184	0.021		
	R	30	22	− 10	4.81	162	0.036		

Anatomical label	Main effect of contingency			Main effect of face perception			Interaction		
	F value	Corrected p-value	Effect size η^2^ (95%CI)	F value	Corrected p-value	Effect size η^2^ (95%CI)	F value	Corrected p-value	Effect size η^2^ (95%CI)
Supramarginal gyrus	1.36	> 1	0.003 (0, 0.02)	0.41	> 1	0.0004 (0, 0.004)	1.84	> 1	0.002 (0, 0.01)
Superior temporal sulcus	3.86	0.35	0.008 (0.0001, 0.03)	1.32	> 1	0.003 (0, 0.02)	0.38	> 1	0.0007 (0, 0.008)
Middle frontal gyrus	**13.6**	**0.005**	**0.03 (0.006, 0.06)**	5.31	0.5	0.007 (0, 0.02)	0.78	> 1	0.0009 (0, 0.009)
	**14.1**	**0.004**	**0.01 (0.004, 0.04)**	1.68	> 1	0.001 (0, 0.009)	3.39	0.45	0.004 (0, 0.009)
Anterior Insula	**10.2**	**0.02**	**0.02 (0.002, 0.05)**	1.40	> 1	0.002 (0, 0.02)	7.51	0.06	0.008 (0.0008, 0.02)
	**10.4**	**0.02**	**0.02 (0.002, 0.04)**	0.30	> 1	0.0003 (0, 0.004)	2.65	0.68	0.003 (0, 0.01)

For each activation peak, the Montreal Neurological Institute (MNI) coordinates (x, y, and z), t-value, cluster size (voxel size = 2 × 2 × 2 mm^3^), corrected *P*, and ROI results are described. Peaks represent the negative contingency effect contrast (i.e., [FN + ON] > [FC + OC]) at *P* < 0.001 (uncorrected), which were corrected according to a family-wise error of *P* < 0.05 based on cluster size. L and R indicate left the (L) and right (R) hemispheres, respectively. The nine right columns provide the ROI analysis results for the recognition phase. The significance threshold of the ROI analysis was Bonferroni-corrected (*P* < 0.05) because multiple comparisons were performed. The F and *P*-values of significant effects are indicated in bold.

*CI* confidence interval, *FC* face contingent, *FN* face non-contingent, *OC* object contingent, *ON* object non-contingent.

We conducted ROI analysis for the recognition phase using six ROIs that demonstrated significant deactivation in voxel-wise analysis of the contact phase. Two-way ANOVA showed significant main effects of contingency in bilateral middle frontal gyri and bilateral anterior insula activations (Table 1, Fig. 3B). There was no significant main effect of face perception or interaction between contingency and face perception. We also conducted voxel-wise analysis to explore whether the contingency effect during the contact phase influenced brain regions other than the ROIs used for analysis of the recognition phase. We observed significant deactivation of the right inferior frontal gyrus—which overlapped with the right middle frontal gyrus in ROI analysis—and bilateral lingual gyri (Table 2, Fig. 3C). These results imply that deactivation of the frontal component of the frontoparietal self-agency network is associated with the effect of action contingency on social perception. Voxel-wise analysis showed a significant contingency × face interaction on activation of the left cuneus, which was presumably derived from greater activation under the FN condition (Table 2, Fig. 3D).

**Table 2:** Brain regions represent a negative contingency effect in the recognition phase.

Anatomical label	MNI coordinates (peak)				t-value	Cluster	
	L/R	x	y	z		Size (voxels)	Corrected p-value
**Main effect of negative contingency effect**							
Inferior frontal gyrus	R	50	14	26	4.58	238	0.009
Lingual gyrus	L	− 22	− 56	− 2	4.40	175	0.034
	R	8	− 74	14	5.34	269	0.005
**Interaction of negative contingency effect**							
Cuneus	L	− 4	− 80	26	4.93	343	0.001

Peaks were obtained from the negative contingency effect contrast (i.e., [FN + ON] > [FC + OC] for the main effect of contingency and [FN − ON] > [FC − OC] the interaction between contingency and face perception). The threshold for significant activation was initially set at *P* < 0.001 (uncorrected), and then corrected to *P* < 0.05 for multiple comparisons based on cluster size. The other details are similar to those presented in Table 1.

**Figure 4:**
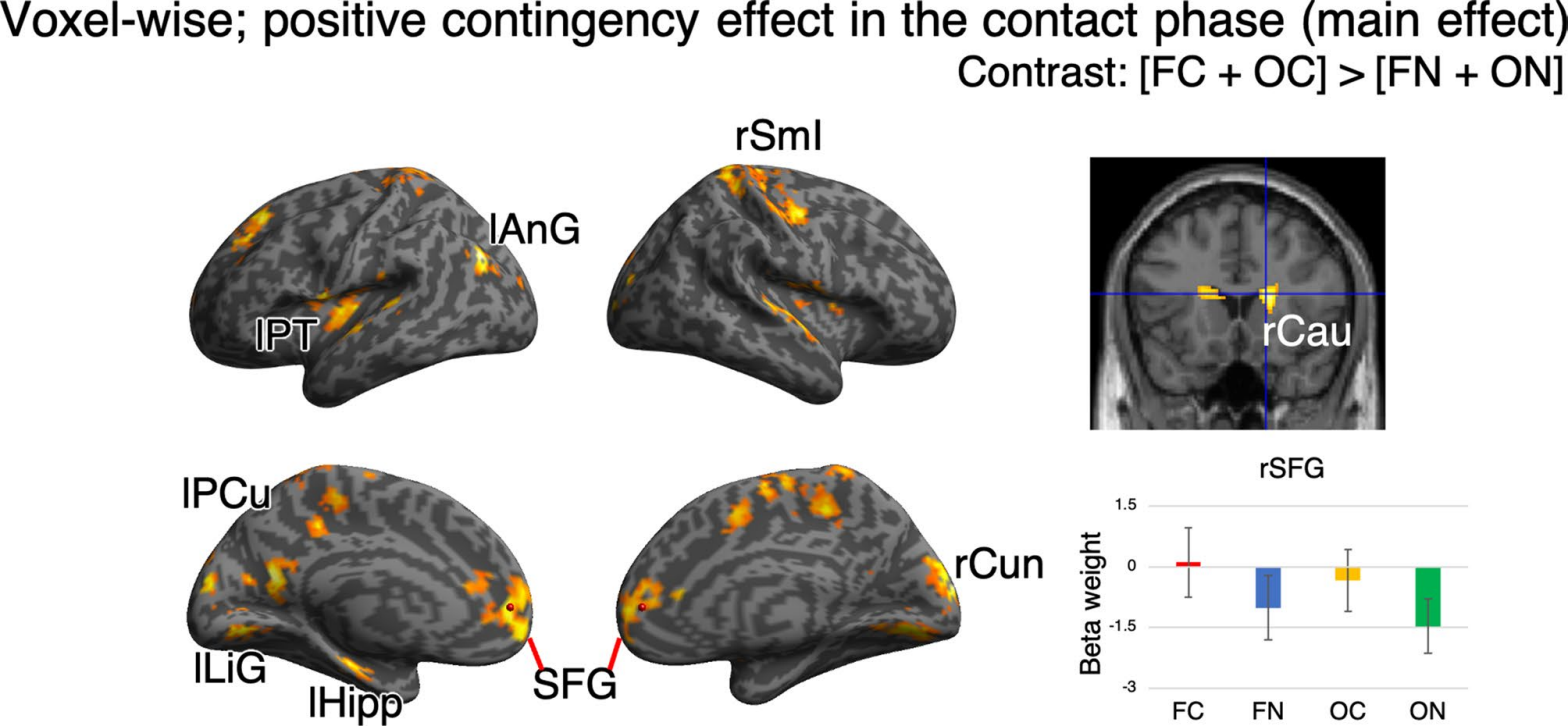
Brain activation representing a positive contingency effect. Brain activation during the contact phase representing a positive contingency effect in voxel-wise analysis. Other details correspond with those in Fig. 3A. *SFG* superior frontal gyrus, *lHipp* left hippocampus, *lAnG* left angular gyrus, *lPT* left planum temporale, *lPCu* left precuneus, *LiG* lingual gyrus, *rSmI* right primary somatosensory cortex, *rCau* right caudate, *rCun* right cuneus.

Analysis of the positive contingency effect (i.e., activation in the [FC + OC] > [FN + ON] contrast) showed significant brain activation in the following regions: bilateral superior frontal gyrus, left hippocampus, left angular gyrus, left planum temporale, left precuneus, left lingual gyrus, right primary somatosensory cortex, right cuneus, right caudate, and right putamen (Table 3, Fig. 4). Activation of these regions is consistent with previous brain activation during contact with a contingent target. Previous studies have reported that contact with contingent targets activated the primary somatosensory cortex, hippocampus, and left angular gyrus^21,22^. In a previous study, the right caudate overlapped with brain regions that were involved in action-contingency-induced changes in liking scores^23^. We did not observe any significant interaction in terms of activation (i.e., [FC − OC] > [FN − ON] contrast), which implies that the action-contingency effect was independent of person-like appearance. For analysis of the recognition phase, we conducted ROI analysis using 11 ROIs that demonstrated significant activation in voxel-wise analysis of the contact phase. Two-way ANOVA showed no significant main effect of contingency or interaction. Subsequent voxel-wise analysis showed no significant main effect of contingency or interaction on activation.

**Table 3:** Brain regions representing a significant positive contingency effect and region of interest (ROI) analysis results.

Anatomical label	MNI coordinates (peak)				t-value	Cluster			
	L/R	x	y	z		Size (voxels)	Corrected p-value		
Superior frontal gyrus	L	− 20	34	36	6.03	2026	< 0.001		
	R	4	54	6	6.75	1665	< 0.001		
Hippocampus	L	− 26	− 20	− 18	5.86	190	0.019		
Angular gyrus	L	− 44	− 72	30	6.61	219	0.010		
Planum temporale	L	− 54	− 32	14	5.26	174	0.027		
Precuneus	L	− 6	− 62	16	6.20	426	< 0.001		
Lingual gyrus	L	− 8	− 74	− 4	5.84	1072	< 0.001		
Primary somatosensory cortex	R	12	− 32	58	6.04	3044	< 0.001		
Cuneus	R	14	− 92	18	6.55	863	< 0.001		
Caudate	R	18	18	16	5.81	370	< 0.001		
Putamen	R	26	− 4	4	5.68	1365	< 0.001		

Anatomical label	Main effect of contingency			Main effect of face perception			Interaction		
	F value	Corrected p-value	Effect size η^2^ (95%CI)	F value	Corrected p-value	Effect size η^2^ (95%CI)	F value	Corrected p-value	Effect size η^2^ (95%CI)
Superior frontal gyrus	2.89	0.99	0.008 (0, 0.03)	0.51	> 1	0.001 (0, 0.01)	1.88	> 1	0.003 (0, 0.02)
	< 0.001	> 1		1.97	> 1		3.30	0.77	
Hippocampus	0.008	> 1	0 (0, 0.0001)	0.25	> 1	0.0005 (0, 0.005)	0.04	> 1	0.0001 (0, 0.001)
Angular gyrus	0.32	> 1	0.0006 (0, 0.008)	4.21	0.54	0.01 (0.0001, 0.05)	1.69	> 1	0.003 (0, 0.01)
Planum temporale	1.27	> 1	0.003 (0, 0.02)	1.80	> 1	0.004 (0, 0.02)	0.89	> 1	0.001 (0, 0.01)
Precuneus	2.80	> 1	0.005 (0, 0.02)	0.47	> 1	0.0009 (0, 0.01)	7.32	0.12	0.008 (0.0006, 0.02)
Lingual gyrus	3.09	0.98	0.002 (0, 0.01)	0.002	> 1	0 (0, 0)	0.58	> 1	0.0005 (0, 0.001)
Primary somatosensory cortex	0.16	> 1	0.0004 (0, 0.004)	0.15	> 1	0.0006 (0, 0.007)	1.79	> 1	0.005 (0, 0.03)
Cuneus	2.87	> 1	0.003 (0, 0.01)	0.15	> 1	0.0004 (0, 0.004)	1.39	> 1	0.002 (0, 0.01)
Caudate	0.66	> 1	0.002 (0, 0.01)	0.04	> 1	0.0001 (0, 0.001)	0.02	> 1	0.0001 (0, 0.0006)
Putamen	2.97	> 1	0.008 (0, 0.03)	0.67	> 1	0.001 (0, 0.01)	0.63	> 1	0.001 (0, 0.009)

Peaks were obtained from the positive contingency effect contrast (i.e., [FC + OC] > [FN + ON]). Other details are similar to those presented in Table 1.

We also investigated whether these neural responses reflected changes in the participants’ subjective liking scores. Correlation analysis showed no significant correlation between liking score changes according to contingency (i.e., difference in liking scores according to contingency; [FC + OC]/2 – [FN + ON]/2) and brain responses in the contact and recognition phases (Supplementary Table 2).

To rule out the possibility that participants did not recognize face avatars as person-like, we conducted additional analyses. Neural activity for the contrast representing the main effect of face perception (i.e., [FC + FN] > [OC + ON]) was examined throughout the contact and recognition phases. The results are presented in Supplementary Fig. 1 and Supplementary Table 3. We observed significant activation in the bilateral superior temporal sulci and right fusiform gyrus, which are related to face processing and socio-cognitive processing^45–48^. These brain regions did not overlap with regions representing the main effect of action contingency during the recognition phase.

## Discussion

In this study, we investigated the effect of action contingency on social perception. We aimed to determine whether action contingency during contact represents recognition of an avatar during subsequent social perception, and whether the effect of action contingency on social perception depends on person-like appearance. Our findings reveal that the effect of action contingency on social perception was encoded as a representation of the avatar. We observed deactivation of the frontal component of the frontoparietal self-agency network (i.e., bilateral inferior-middle frontal gyri and insula) during the recognition phase. However, activation of reward-related regions during the contact phase was not replicated during the recognition phase. Our results also show that the effect of action contingency on neural responses was independent of person-like appearance. Brain activation representing the main effect of action contingency on social perception during the recognition phase, i.e., the frontal component of the frontoparietal network, was observed regardless of person-like appearance.

Our findings related to the first issue (i.e., brain activation during the contact phase was replicated in the recognition phase) imply that the action-contingency effect on social perception is observed at the level of the representation of contacting partners. We observed deactivation of the frontal component of the frontoparietal network during the recognition phase. These regions participate in action monitoring, as well as the association between a person’s own actions and sensory input; these regions are deactivated on receipt of sensory input that is not derived from a person’s own actions^4,26,27^. These regions are also deactivated during interactions with others whose actions are contingent on a person’s own actions^22,23^. Notably, we observed deactivation of these action-perception-related regions even when the participants looked at static images during the recognition phase, implying that the action-contingency effect was encoded as a representation of contacting partners. Finally, activation of these regions reflects social perception, which is important for group membership. A recent meta-analysis reported reduced activation of these regions for in-group members compared with out-group members^49^; importantly, this reduced activation was limited to the frontal regions. In this context, our findings imply that the frontal component is involved in processes in which the influence of contingency related to others’ actions during contact contributes to subsequent social perception.

Our findings related to the second issue (i.e., deactivation of frontoparietal regions was observed as the main effect of action contingency) support the hypothesis that the action-contingency effect is independent of person-like appearance. We found a significant main effect of action contingency on subjective liking scores, regardless of face-like appearance, implying that person-like appearance is not always needed in social perception. We did not observe a significant interaction between action contingency and face perception, which would have been seen if the action-contingency effect was dependent on person-like appearance. However, we did observe an effect of person-like appearance on social perception; activation of brain regions related to face processing and socio-cognitive processing^45–48^ represented the main effect of face perception. The lack of overlap between brain activation representing the main effect of action contingency and that representing the main effect of face perception also implies the independence of the action-contingency effect. Our findings support the hypothesis that the action-contingency effect on social interaction shares a mechanism with the sense of self-agency^10,20^; these effects are independent of person-like appearance. These findings also support the theory that person-like appearance and action contingency contribute independently to socio-emotional development^42^. However, deactivation of frontoparietal regions may also represent non-motor self-agency, such as inferential self-agency (i.e., inferring whether an outcome is derived from one’s own behavior)^50^, and thus, the detailed relationships between the action-contingency effect and self-agency remain unclear.

Changes in subjective liking scores were not associated with action-contingency-induced neural responses; this lack of association may have been related to the methodology applied in this study. Previous fMRI studies found no relationship between frontoparietal deactivation and liking indices^21–23^; in this respect, our findings are not inconsistent with previous studies. In addition, previous fMRI studies of the association between liking indices and reward-related regions investigated liking indices after contact, but did not compare indices before and after contact^21,23^. Thus, the observed correlation between reward-related regions and liking indices may not have reflected changes in social perception caused by action contingency. There are many differences among studies that investigated the action-contingency effect on social perception, such as task design (e.g., drumming in rhythm with partners vs. interacting with moving partners), liking indices (e.g., evaluating questionnaire scores after contact vs. before and after contact), and neural responses (e.g., contrasts such as [contact with a contingent partner vs. looking at a fixation point] and [contact with a contingent partner vs. contact with a non-contingent partner]). These differences may explain inconsistencies among previous fMRI studies, for example in activation related to reward-related regions [c.f. Cacioppo et al. (2014) and Kokal et al. (2011)], as well as our own negative results. Future studies of the association between subjective liking scores and action-contingency-induced neural responses should consider these methodological differences.

The face-dependent contingency effect observed in the recognition phase presumably reflects a task-specific attention effect, rather than an effect on social perception. Based on the negative-contingency-effect contrast, we observed significant activation of the left cuneus in the recognition phase; this represented the interaction between contingency and face perception. The cuneus is the visual-processing region^51^; behavioral data imply that participants required considerable time to rate the FN-condition avatar. The lack of a significant correlation between changes in liking scores according to the contingency effect and cuneus activation implies that activation of the cuneus reflects the duration of attention given to the avatar.

In conclusion, the findings of this study imply that the action-contingency effect occurs at the level of representation of contacting partners, independent of person-like appearance. Our results imply that the independent action-contingency effect on social perception is reflected by deactivation of the frontal component of the frontoparietal self-agency network, providing novel evidence that the action-contingency effect on social perception is an extension of action self-agency. The current results have theoretical implications for the potential developmental roles of action contingency processing in a wider range of human social faculties^52–54^. The findings of this study also have theoretical implications for the development of communicative robots; consideration of the independent effects of visual features such as face-like appearance and action contingency could lead to the development of interactive robots^2,6,43^.

This study had several limitations. First, because our participants were adults, the potential implications of our findings for the development of sociality remain entirely speculative. Second, we did not evaluate the duration of the action-contingency effect. We observed deactivation only in the frontal component of the frontoparietal self-agency network immediately after contact; however, behavioral evidence of improved social perception in a previous study implies that the effect lasts longer^23^. Additional studies are needed to investigate the persistence of the effect of action contingency on social perception at the neural level.

## Methods

### Ethical approval

This study was approved by the Ethics Committee of Tohoku University Graduate School of Medicine, Japan (2016-1-272). We obtained informed consent from all participants and conducted the study in accordance with the Declaration of Helsinki.

### Participants

In total, 41 participants were recruited from the undergraduate and graduate schools of Tohoku University. The main purpose of this study is to investigate brain activation related to the action-contingency effect on social perception. Thus, the sample size was determined according to task-based fMRI studies conducted previously at our institution (e.g., Kageyama et al., 2019^55^ and Motoki et al., 2019^56^). Three participants were excluded because of machine errors during recording, and seven were excluded because of excessive head movements (i.e., > 7 mm). Thus, we analyzed behavioral and brain data from 31 participants (15 men, age [mean ± standard deviation] = 20.68 ± 1.47 years).

### Task design

The experiment consisted of two phases: contact (Fig. 1A) and recognition (Fig. 1B). In the contact phase, participants made contact with the avatars, which appeared to be unrelated to the task. After an eye-fixated resting period (randomly set at 2, 3, or 4 s), an avatar was presented above the fixation point for 2 s. Participants were instructed to observe the fixation point and to press the right or left button when the fixation point became brighter. The participants were also instructed not to consider the avatar when pressing the button. Immediately after the button was pressed, a red square appeared to the right or left side (direction of the button pressed) of the fixation point. Thus, the appearance of the red square signaled to the participants that they had successfully pressed the button. The button press cue was presented 1 s after avatar presentation. Participants were required to press the button within 0.5 s after the button-press cue was presented. Contingent avatars (FC and OC) moved in the direction of the button press, whereas non-contingent avatars (FN and ON) moved randomly, regardless of the participant’s choice, before or after the button press (Fig. 1A). Participants completed 12 trials in the contact phase.

Avatars were colored spheres with four black geometric shapes on their surface (Fig. 1C). We arranged the four black geometric shapes to simulate a face during the creation of face-like avatars (FC and FN; Fig. 1C). In contrast, geometric shapes were placed in a line to avoid a face-like appearance during the creation of non-face-like avatars (OC and ON; Fig. 1C). Thus, there were four types of avatars (two contingency conditions × two face-perception conditions), which were distinctly color-coded (red, blue, yellow, and green) to ensure that participants could identify the avatar’s type. The assignment of colors to each condition was counterbalanced across participants.

During the recognition phase, the avatar was presented as a static image and participants were asked to evaluate their subjective liking score. Based on previous studies, we used three questionnaire items to investigate the participants’ perception of each avatar^21,43,57^: “Do you like the avatar?”, “Do you think the avatar is nice?”, and “Do you want the avatar?”. In each trial, participants observed the fixation point for 2.5, 4.5, or 6.5 s. Then, one static avatar was displayed for 1 s, while one of the three questionnaire items was presented for 2.5 s (Fig. 1B). Therefore, participants completed 12 trials (4 avatars × 3 questionnaire items) in a random order. Participants answered each questionnaire item using an eight-point Likert-like scale, from 1 (not at all) to 8 (very much), by pressing the button.

We performed four blocks of contact and recognition phases during one session (Fig. 1D). We repeated the session five times (i.e., participants completed five sessions) because we expected that the action-contingency effect would gradually increase with contact repetition; however, this enhancement was not observed (see Results for details). To investigate participants’ baseline perception of each avatar, we conducted one block of the recognition phase before the fMRI sessions (“Pre rating” in Fig. 1D).

### Experimental procedures

Before the experiment, participants rated their baseline perception of each avatar (Fig. 1C). Participants practiced the experimental procedures in one block of the contact and recognition phases outside the MRI scanner. The practice session used a combination of avatar color and movements similar to parameters in the actual experiment. Participants completed the practice session before they rated baseline perception of each avatar, which might have influenced the baseline ratings. However, since participants made contact with each avatar only three times in the practice session, the contingency effect of practice was expected to be negligible.

After the practice session and baseline rating, participants entered the MRI scanner. The task was projected onto a semi-lucent screen behind the coil of the MRI scanner using PsychoPy 2 ver. 1.83.0^58,59^. Participants viewed the task through a mirror mounted on the head coil. Participants placed each hand on a keypad with four buttons. During the contact phase, participants pressed the button using their left or right index finger. During the recognition phase, participants answered the questions using eight buttons (i.e., from right little finger to left little finger). Participants were allowed approximately 30 min of rest twice between sessions (i.e., between sessions 2 and 3, and between sessions 4 and 5).

### fMRI data acquisition

Images were acquired using a 3 T MRI scanner (Philips Achieva, Best, Netherlands). Whole-brain fMRI data were acquired using T2*-weighted gradient echo-planar imaging. In total, 38 slices of gradient-echo images (echo time = 30 ms, flip angle = 85°, slice thickness = 3 mm, slice gap = 0.5 mm, field of view = 192 mm, and matrix size = 64 × 64) covering the whole brain were acquired with a repetition time of 2500 ms. A structural whole-brain image was acquired using magnetization-prepared rapid-acquisition gradient-echo, with the following parameters: repetition time = 6.7 ms, echo time = 3.1 ms, field of view = 192 mm, number of slices = 162, and slice thickness = 1 mm.

### Behavioral data analysis

We calculated each participant’s liking score as the summed score of three questions (i.e., “Do you like the avatar?”, “Do you think the avatar is nice?”, and “Do you want the avatar?”). We obtained the liking scores of the four avatars separately for baseline and sessions 1–5. Each session was composed of four blocks, and the liking score of each session was the mean of four blocks.

We expected that liking scores would gradually increase during the fMRI sessions; however, they rapidly increased between baseline and session 1 (Fig. 2A). Repeated measures three-way ANOVA showed that liking scores were not affected by session (see the Results section for details). Therefore, we compared the liking scores before the experiment (“Pre rating” in Fig. 1D) with the mean liking scores of the five experimental sessions (S1–S5 in Fig. 1D). The fMRI data for the five experimental sessions were pooled for analysis.

Then, we analyzed the behavioral and fMRI data by pooling data from the five fMRI sessions. We calculated the liking score difference by subtracting the liking score before the experiment from the mean score of sessions 1–5 (i.e., [(S1 + S2 + S3 + S4 + S5)/5] − [Pre]; Fig. 2A). Then, we performed 2 × 2 (2 contingency factors × 2 face perception factors) repeated measures two-way ANOVA (Fig. 2B) using R software (ver. 3.3.3; https://www.R-project.org/). Statistical significance was set at *P* < 0.05. The effect sizes were examined, and 95% CIs were calculated using the bootstrapping method (2,000 iterations) following bias correction and percentile acceleration.

### fMRI data analysis

We conducted preprocessing using statistical parametric mapping software (SPM12; Wellcome Department of Imaging Neuroscience; Institute of Neurology, London, United Kingdom) and MATLAB. Preprocessing included correction for head motion, adjustment of acquisition time across slices, co-registration to anatomical image, spatial normalization using the anatomical image and the Montreal Neurological Institute template, and smoothing using a Gaussian kernel with full-width at half-maximum of 6 mm.

Data from the contact and recognition phases were analyzed using a conventional two-level approach within SPM12^60^. In the first-level analysis, we constructed a within-subject voxel-wise multiple regression model. To identify brain activity related to each condition (i.e., FC, FN, OC, and ON), we modeled 40 regressors (four conditions × two phases × five sessions). The event was modeled at the onset of avatar presentation (i.e., 2 s of avatar presentation during the contact phase and 1 s of static avatar presentation during the recognition phase). The estimated parameters of head movements obtained by preprocessing were also modeled as regressors to exclude the effect of head motion as a covariate of no interest. A high-pass filter (cutoff of 128 s) was applied.

In the second-level analysis, we separately analyzed the contact and recognition phases. We investigated the main effect and interaction of the 2 × 2 experiment using the estimated image of each participant. Our behavioral results showed that the participants’ subjective liking scores did not change significantly during sessions (Fig. 2A); therefore, we pooled brain activities from the five sessions. First, we investigated the main effect of contingency and the interaction between contingency and face perception during the contact phase. We investigated the negative contingency effect (i.e., N > C) and the positive contingency effect (i.e., C > N) by creating separate contrasts; the negative-effect contrasts were [FN + ON] > [FC + OC] (main effect contrast) and [FN − ON] > [FC − OC] (interaction contrast), whereas the positive-effect contrasts were [FC + OC] > [FN + ON] (main effect contrast) and [FC − OC] > [FN − ON] (interaction contrast). We performed one-sample t-tests and set the threshold for significant activation at *P* < 0.001 (uncorrected), which was corrected to *P* < 0.05 for multiple comparisons using the cluster size.

Next, to examine the effect of action contingency on subsequent social perception, we investigated brain activity during the recognition phase. To investigate whether our findings could be replicated during the recognition phase, we performed ROI analysis using significant regions identified during the contact phase. We performed two-way ANOVA to investigate the main effect of contingency and the interaction between contingency and face perception. We identified 6 and 11 significant ROIs from the contact phase using the negative- and positive-contingency-effect contrasts, respectively. Bonferroni correction was applied using the number of ROIs, with statistical significance set at *P* < 0.05 (Bonferroni-corrected, α = 0.05/6 and 0.05/11 for negative- and positive-contingency-effect contrasts, respectively). In addition, to explore whether the contingency effect during the contact phase influenced brain regions other than the ROIs identified during the recognition phase, we performed a voxel-wise analysis approach. We used the same contrasts as in the contact phase and performed one-sample t-tests. The threshold for significant activation was initially set at *P* < 0.001 (uncorrected), then corrected to *P* < 0.05 for multiple comparisons using the cluster size.

We performed correlation analysis to investigate the relationships between liking-score changes and brain activity; we did not observe any changes in liking scores during the sessions (see Results for details). Then, we calculated the contingency effect on changes in liking scores by subtracting the liking scores for contingent conditions from those for non-contingent conditions (i.e., [FC + OC]/2 – [FN + ON]/2). We investigated correlations between changes in liking scores and two types of brain activity during the recognition phase: brain activity obtained from the ROI analysis and brain activity obtained from voxel-wise analysis of the recognition phase. Bonferroni correction was used for correlation analysis, and the statistical significance was set at *P* < 0.05.

The effect sizes of two-way ANOVA were examined, and their 95% CIs were calculated using the bootstrapping method (2,000 iterations) following bias correction and percentile acceleration (Tables 1 and 3). The results of correlation analysis between brain activation and liking scores are presented in Supplementary Table 2. The effect sizes of voxel-wise analysis were not examined because there is no established method to calculate the effect size.

## Supplementary Information

Supplementary Information.

## Data Availability

Data obtained in the current study are available from the corresponding author (YH) upon reasonable request, including a project outline.
