# Supplementary material for: The effect of action contingency on social perception is independent of person-like appearance and is related to deactivation of the frontal component of the self-agency network
Source: Sci Rep. 2022 Oct 15;12:17326. doi: 10.1038/s41598-022-22278-x (PMC9568912; doi:10.1038/s41598-022-22278-x)
Supplement: Supplementary file 1 — Supplementary Information. [file 41598_2022_22278_MOESM1_ESM.pdf]

The effect of action contingency on social perception is independent of person-like appearance and is related to deactivation of the frontal component of the self-agency network.

Yumi Hamamoto [a,b]*, Yukiko Takahara[b], Kelssy Hitomi dos Santos Kawata[a,b], Tatsuo Kikuchi[a,b], Shinsuke Suzuki[a,c,d], Ryuta Kawashima[a], Motoaki Sugiura[a,e]

a. Institute of Development, Aging and Cancer, Tohoku University, Sendai, Japan

b. School of Medicine, Tohoku University, Sendai, Japan

c. Frontier Research Institute for Interdisciplinary Science, Tohoku University, Sendai, Japan

d. Centre for Brain, Minds and Markets, Department of Finance, Faculty of Business and Economics, The University of Melbourne, Australia

e. International Research Institute of Disaster Science, Tohoku University, Sendai, Japan

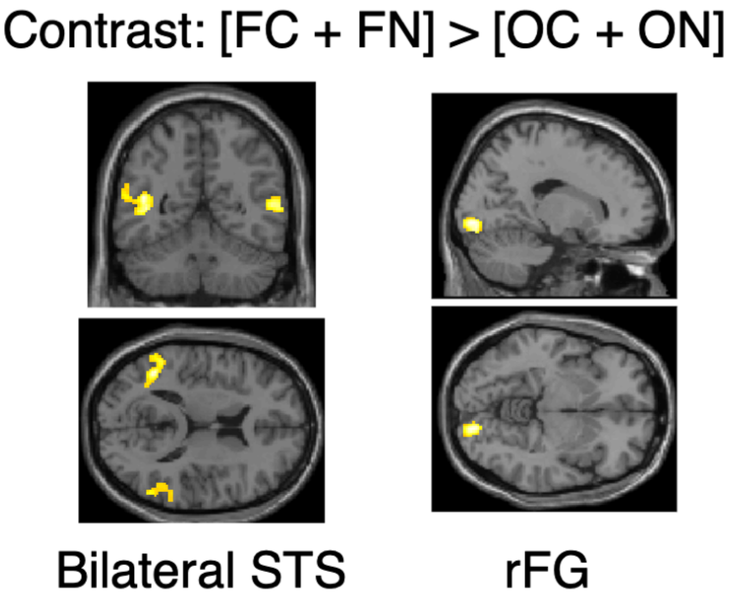

Supplementary Figure 1. Brain activation representing the main effect of face perception.

Brain activation during the contact and recognition phases indicating a main effect of face perception. The threshold was set at $P < 0.001$ (uncorrected) and corrected to $P < 0.05$ based on cluster size. These brain regions participate in face perception [46, 48]. In addition, the superior temporal sulci participate in socio-cognitive processing [45, 47]. FG = fusiform gyrus, STS= superior temporal sulcus.

Supplementary Table 1. Button preference and reaction time for each condition.

	FC	FN	OC	ON	Main effect of contingency			Main effect of face perception			Interaction		
					F value	p value	Effect size η^2 (95%CI)	F value	p value	Effect size η^2 (95%CI)	F value	p value	Effect size η^2 (95%CI)
Right button (SD)	30.5 (7.11)	30.5 (7.86)	30.0 (7.89)	30.2 (6.90)	0.08	0.79	0.0002 (0, 0.003)	0.62	0.44	0.001 (0, 0.01)	0.28	0.60	0.0004 (0, 0.006)
Left button (SD)	25.7 (7.79)	24.8 (8.44)	26.3 (7.11)	25.3 (7.33)	1.03	0.32	0.003 (0, 0.04)	0.62	0.44	0.001 (0, 0.01)	0.007	0.94	0 (0, 0.0001)
Failed (SD)	3.81 (2.83)	4.71 (3.30)	4.03 (3.31)	4.45 (2.86)	2.55	0.12	0.01 (0, 0.05)	0.005	0.95	0 (0, 0)	0.42	0.52	0.002 (0, 0.02)

RT in the contact phase (SD)	0.38 (0.09)	0.38 (0.10)	0.38 (0.10)	0.37 (0.10)	2.17	0.14	0.0003 (0, 0.001)	6.78	0.009	0.0008 (0, 0.002)	0.29	0.59	0 (0, 0.0004)
RT in the recognition phase (SD)	1.18 (0.42)	1.24 (0.43)	1.22 (0.45)	1.20 (0.43)	2.99	0.08	0.0002 (0, 0.001)	0.54	0.46	0 (0, 0.002)	31.5	<0.001	0.002 (0.0008, 0.004)

The mean reaction times during the contact and recognition phases are shown. The counts of right and left button presses, as well as failed button presses, during the contact phase are also shown. Numbers in parentheses indicate 95% CIs. RT = reaction time; SD = standard deviation.

Supplementary Table 2. Correlations between changes in liking scores according to contingency and brain activity during the contact and recognition phases.

Brain regions	Correlation coefficient (95%CI)
Contact phase (negative contingency effect)	
Left supramarginal gyrus	0.28 (-0.09, 0.66)
Right superior temporal sulcus	0.21 (-0.16, 0.59)
Left middle frontal gyrus	0.25 (-0.12, 0.62)
Right middle frontal gyrus	0.39 (0.05, 0.79)
Left anterior insula	0.36 (0.002, 0.74)
Right anterior insula	0.33 (-0.03, 0.71)
Contact phase (positive contingency effect)	
Left superior frontal gyrus	0.04 (-0.33, 0.41)
Right superior frontal gyrus	-0.17 (-0.54, 0.20)
Left Hippocampus	-0.14 (-0.51, 0.23)
Left angular gyrus	0.19 (-0.18, 0.56)
Left planum temporale	0.06 (-0.32, 0.43)
Left precuneus	0.16 (-0.21, 0.53)

Left lingual gyrus	-0.07 (-0.44, 0.30)
Right primacy somatosensory cortex	-0.11 (-0.48, 0.26)
Right cuneus	-0.14 (-0.51, 0.23)
Right caudate	-0.25 (-0.63, 0.11)
Right putamen	0.02 (-0.35, 0.39)
Recognition phase (negative contingency effect)	
Left middle frontal gyrus (ROI)	0.03 (-0.34, 0.40)
Right middle frontal gyrus (ROI)	0.03 (-0.34, 0.40)
Left anterior insula (ROI)	0.13 (-0.24, 0.50)
Right anterior insula (ROI)	0.09 (-0.28, 0.45)
Right inferior frontal gyrus (voxel-wise)	-0.05 (-0.42, 0.32)
Left lingual gyrus (voxel-wise)	0.02 (-0.35, 0.39)
Right lingual gyrus (voxel-wise)	-0.20 (-0.22, 0.52)

Simple correlations between changes in liking scores according to contingency and brain activity were investigated. The contact phase included 6 and 11 correlations in the negative- and positive-contingency-effect contrasts, respectively; the recognition phase included seven correlations in the negative-contingency-effect contrast [four from region of interest (ROI) analysis and three from voxel-wise analysis]. The statistical

significance was set at Bonferroni-corrected $P < 0.05$ using the numbers of correlations in each contrast and phase. No correlation was significant after Bonferroni correction. Numbers in parentheses indicate 95% confidence intervals (CIs).

Supplementary Table 3. Brain regions representing a main effect of face perception during the contact and the recognition phases.

Anatomical label	MNI coordinates (peak)				t-value	Cluster	
	L/R	x	y	z		Size (voxels)	Corrected p-value
Superior temporal sulcus	L	-44	-54	14	5.69	393	0.001
	R	56	-58	10	5.96	269	0.004
Fusiform gyrus	R	18	-90	-6	5.78	215	0.01

Peaks were obtained from the negative-contingency-effect contrast (i.e., [FC + FN] > [OC + ON] for the main effect of face perception. For each activation peak, the Montreal Neurological Institute (MNI) coordinates (x, y, and z), t-value, cluster size (voxel size = $2 \times 2 \times 2$ mm^3), corrected *P* and effect size are shown. The threshold for significant activation was initially set at $P < 0.001$ (uncorrected), and then corrected to $P < 0.05$ for multiple comparisons based on cluster size. Numbers in parentheses indicate 95% CIs.
